# Supplementary figures and images for: Increased hepatic interleukin-1, arachidonic acid, and reactive oxygen species mediate the protective potential of peptides shared by gut cysteine peptidases against Schistosoma mansoni infection in mice
Source: PLoS Negl Trop Dis. 2023 Mar 15;17(3):e0011164. doi: 10.1371/journal.pntd.0011164 (PMC10042345; doi:10.1371/journal.pntd.0011164)

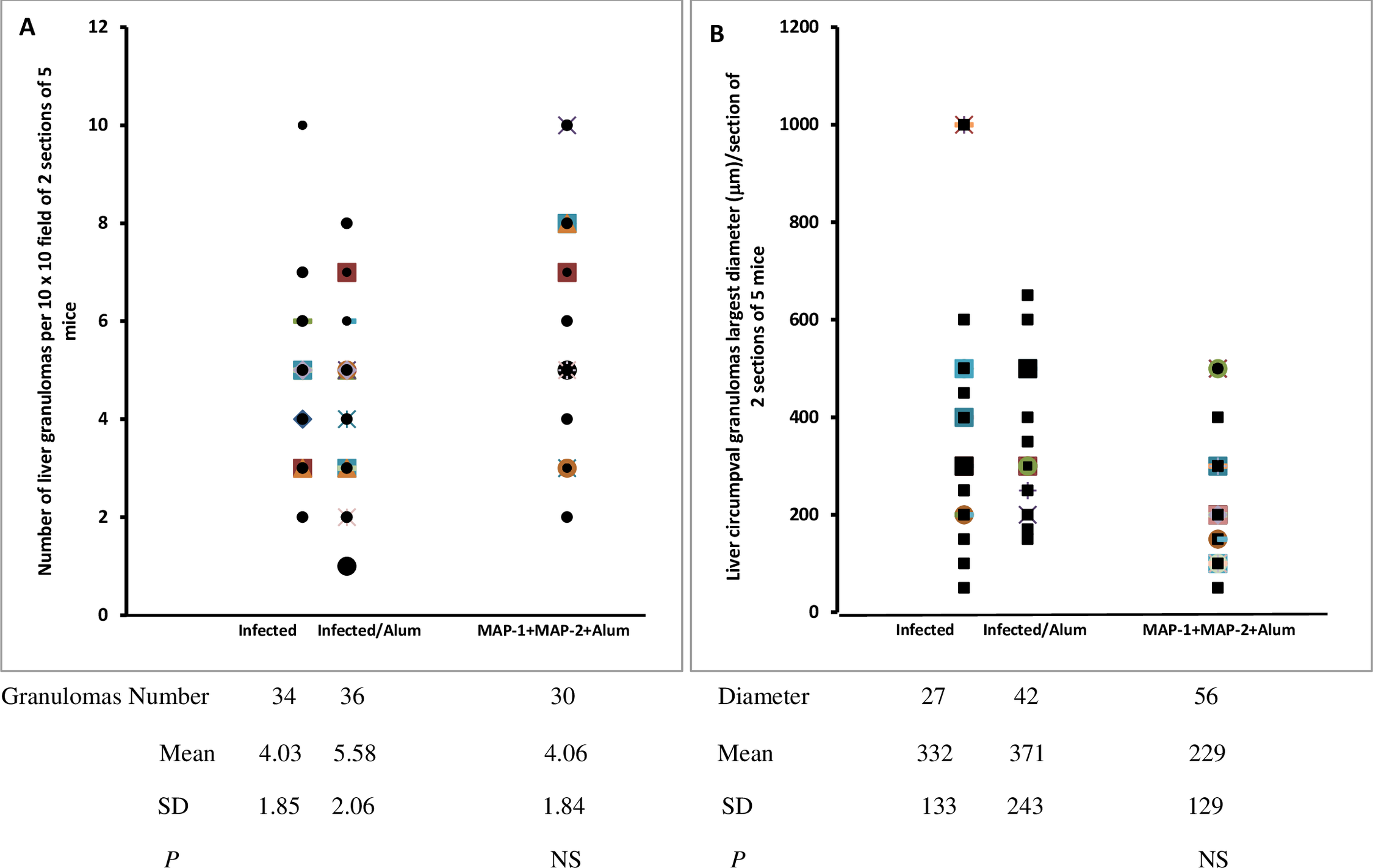

Supplement: S1 Fig — Liver granulomas number (A) and diameter (B) were evaluated 7 wks post challenge infection. Statistical (Mann-Whitney) differences between immunized and infected control mice are not significant (NS). (TIF) [file pntd.0011164.s001.tif]

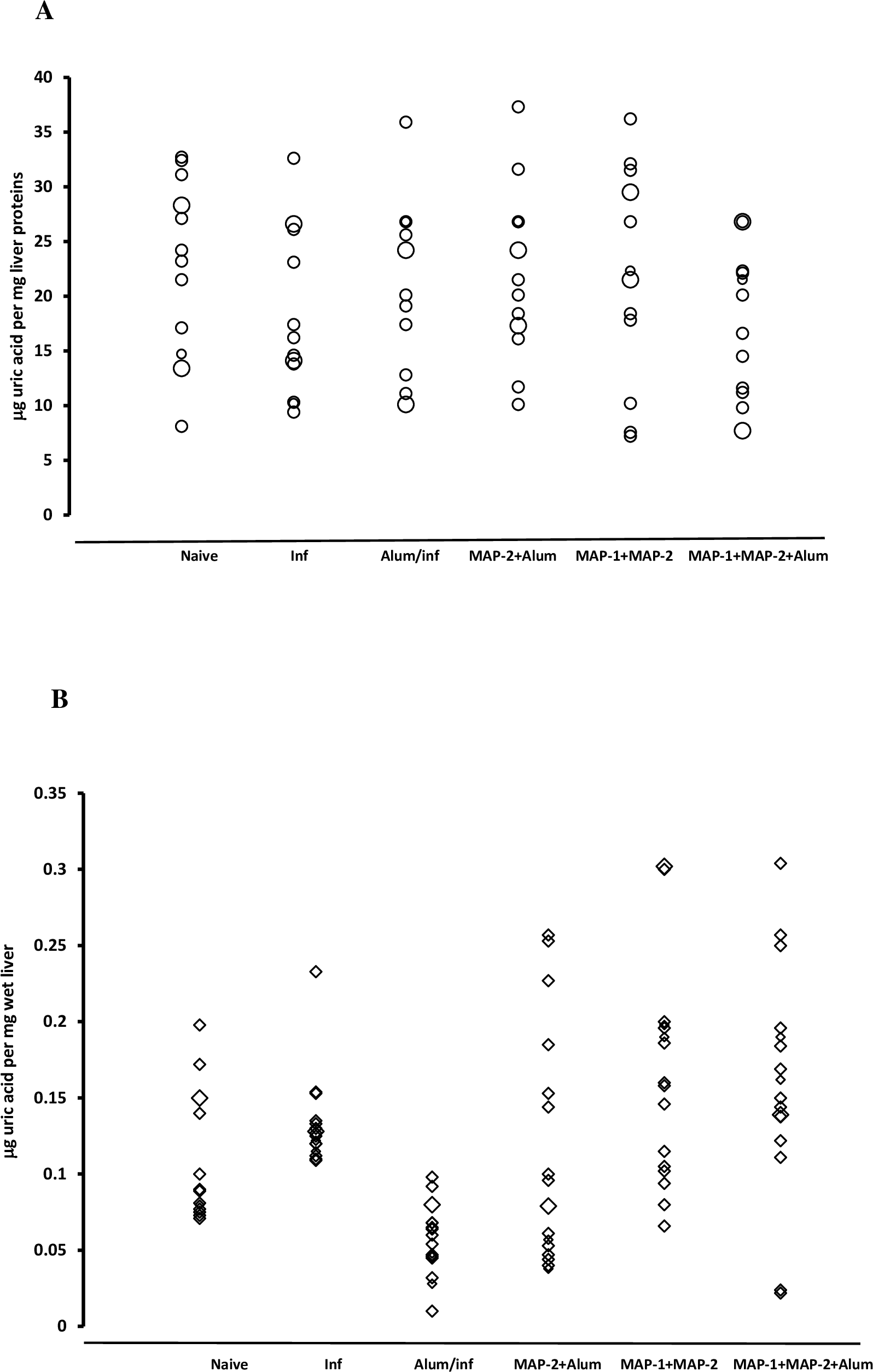

Supplement: S2 Fig — (A) Columns represent μg uric acid/mg liver proteins in 5–8 individual mice/group. Liver Triton X-100 extracts were assayed for uric acid content in duplicate 50 μg protein samples per well using in parallel two separate Uric Acid Assay Kits. Similar results were obtained and were, therefore, pooled. No significant differences between groups were recorded as assessed by ANOVA. (B) Columns represent μg uric acid/mg wet liver in 5–8 individual mice per groups. Results of uric acid content (μg per mg wet liver) with assays of 2.5 and 5 μL/well were similar, and, therefore, pooled. No significant differences between naïve, infected, and MAP-immunized groups were recorded as assessed by ANOVA. (TIF) [file pntd.0011164.s002.tif]

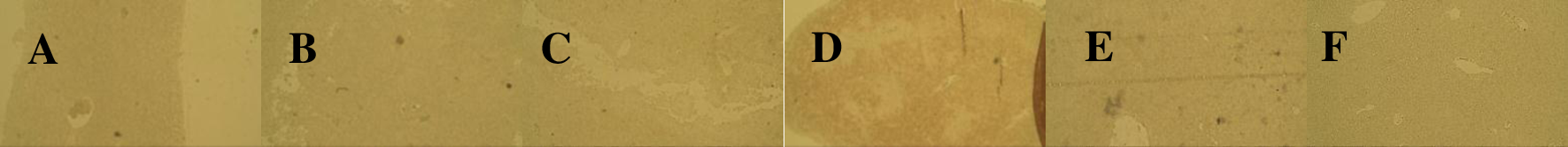

Supplement: S3 Fig — Liver sections of each of 3 naïve (A); infected (B); alum/infected (C); and MAP-2 + alum (D), MAP-1 + MAP-2 (E), and MAP-1 + MAP-2+ alum- (F) immunized mice were reacted with 0.5 μg horseradish peroxidase-linked polyclonal antibody to ARA (MyBioSource, MBS2051576) overnight at 10°C. The reaction was visualized with Dako Liquid DAB + Substrate Chromogen System. Figures shown are representative of the consistently recorded reactivity for each mouse group on day 23 post S. mansoni infection. x100. (TIF) [file pntd.0011164.s003.tif]
