# Supplementary material for: Increased hepatic interleukin-1, arachidonic acid, and reactive oxygen species mediate the protective potential of peptides shared by gut cysteine peptidases against Schistosoma mansoni infection in mice
Source: PLoS Negl Trop Dis. 2023 Mar 15;17(3):e0011164. doi: 10.1371/journal.pntd.0011164 (PMC10042345; doi:10.1371/journal.pntd.0011164)
Supplement: Attachment — Submitted filename: RESPONSE TO PLOS REVIEWERS.docx [file pntd.0011164.s004.docx]

RESPONSE TO PLOS REVIEWERS

Reviewer's Responses to Questions

<b>Key Review Criteria Required for Acceptance?</b></br></br>
As you describe the new analyses required for acceptance, please consider the following:</br></br>

<b>Methods</b></br></br>
-Are the objectives of the study clearly articulated with a clear testable hypothesis stated?</br>
-Is the study design appropriate to address the stated objectives?</br>
-Is the population clearly described and appropriate for the hypothesis being tested?</br>
-Is the sample size sufficient to ensure adequate power to address the hypothesis being tested?</br>
-Were correct statistical analysis used to support conclusions?</br>
-Are there concerns about ethical or regulatory requirements being met?</br></br>

Reviewer #1: (No Response)

Reviewer #2: The two authors of this story present a study in a mouse model that aims to map a highly relevant clinical problem. A vaccine against schistosomes is one of the most important vaccines to be developed, and very difficult due to the complexity of the pathogen and its developmental stages. The vaccine combination MAP-2 in combination with alum and/ or MAP-1 resulted in a 60% worm burden reduction of a challenge infection, which was explained by the authors with significantly increased levels of IL-1 ARA and ROS in the liver.

The objectives of the study are clearly stated. However, **why an older formula had to be modified is just stated but not justified.** It would be nice to hear about expected advantages.

**Reply. Thank you for every statement and query. The older formula is superb but involves three full-length, enzymatically active, recombinant enzymes devised by Prof. Charles Shoemaker and Prof. John P. Dalton who are interested in issues other than the schistosomiasis vaccine. Developing countries, including Egypt, lack the expert science and technology to deliver these molecules, even on a laboratory scale. Production of a single, short, recombinant antigen is, however, feasible. The advantages are still not clear, but that issue is pursued as we have no other alternative or choice.**

The study design, including sample sizes, is appropriate to measure the success of the vaccine combination on a challenge infection.

**Reply. Thank you once again.**

Reviewer #3: The Methods are complete but could be written much more succinctly since these have been reported in several previous manuscripts by the authors

**Reply. Each sentence in the Methods section was checked and it was found that details published previously involve arachidonic detection. However, ARA was previously detected using competitive ELISA, while we have now found that capture ELISA gives more reproducible results, and had to be described in great detail. Detection by histochemistry was previously well described and only newly used antibody and chromogen are now mentioned.**

--------------------

<b>Results</b></br></br>
-Does the analysis presented match the analysis plan?</br>
-Are the results clearly and completely presented?</br>
-Are the figures (Tables, Images) of sufficient quality for clarity?</br></br>

Reviewer #1: (No Response)

Reviewer #2: The results are somewhat disorganized, the graphs are formatted differently, and some of the X-axis labels are missing. Different colors and fonts reduce the clear representation. It appears as if a prefinal version has been submitted.

**Reply: We apologize for these flaws, which have been amended in all Graphs and Figures, including the Supplementary Figures.**

Reviewer #3: The data is interesting but presented very poorly in graphs, figures and Tables **that are not easy to follow.** Much of this data should be placed in the Supplementary data. The text in this section should be more focused on the more relevant results.

**Reply: We apologize for these flaws, which have been amended in all graphs and figures. The text, relevance of Figures, display of Tables have been thoroughly revised and amended.**

<b>Conclusions</b></br></br>
-Are the conclusions supported by the data presented?</br>
-Are the limitations of analysis clearly described?</br>
-Do the authors discuss how these data can be helpful to advance our understanding of the topic under study?</br>
-Is public health relevance addressed?</br></br>

Reviewer #1: (No Response)

Reviewer #2: The conclusion of the abstract "A major mechanism of schistosomiasis vaccine protective potential is now clarified." is not supported by the data. The effect of reduction of worm burden is associated with .... or maybe due to...., elevation of expression of three genes in the liver does not represent a major mechanism.
Limitations and public health relevance are not described.

**Reply: These important concerns have been taken into close consideration, and the Abstract conclusion amended, and changes red-highlighted.**

Reviewer #3: The Discussion followed by Conclusion is again very voluminous and could be reduced significantly.

**Reply: The Discussion and Conclusion were thoroughly revised and every unnecessary or irrelevant aspect deleted, and changes red-highlighted.**

<b>Editorial and Data Presentation Modifications?</b></br><br/>

Use  this section for editorial suggestions as well as relatively minor modifications of existing data that would enhance clarity. If the only modifications needed are minor and/or editorial, you may wish to recommend “Minor Revision” or “Accept”.

Reviewer #1: (No Response)

Reviewer #2: Given the high relevance of the study, vaccines are urgently needed, and given the expert status of the authors, this study disappoints because of the poor quality of expression, language, and presentation. It appears that a prefinal version was submitted. The manuscript urgently needs revision, preferably with the assistance of a native speaker. The abstract needs to be revised since important results are missing.

**Reply: Heartfelt thanks for these criticisms, which have helped to improve the manuscript as they were all taken into the closest considerations and changes red-marked. The abstract was revised and important results displayed within the 300 words maximum allowed.**

The following are just a few examples of typos. The expression needs to be revised as well:
-L77: Revision of the phrase is necessary	**Reply: The sentence was deleted.**
-L138: granuloma numbers **Reply: Replaced by egg granulomas numbers. With thanks.**

-L149: Are the following tests performed with fresh liver tissue? **Reply: YES.**

-L155: better reaction tubes	**Reply: Done with thanks.**
-L171: Arachidonic acid ...assay? **Reply: Done, and changes red-highlighted.**

-L172: Free ARA abbreviation and later L180 Arachidonic acid. **Reply: Because it is at the beginning of a paragraph and a sentence, following a period.**

-L217: Which results justify the conclusion of transit to exit points? **Reply: Thanks are due to the eminent reviewer for this remark, as the results really validating and documenting this conclusion are shown only in Table 2 and Figure 3. Accordingly, the sentence was made clearer and moved to the end of the text of Results section: Parasitological parameters, and changes red-marked.**

-L241: This line is empty. Are there no values? **Reply: No reduction percentage is displayed when the differences are not statistically (Mann-Whitney) different. The line was deleted from the Table with thanks.**

-Fig 1: Labeling of x-axis. **Reply: Done with thanks.**
-L 267 and 268: Revision of the phrase is necessary. **Reply: The results of the previous article [11] render us unable but to stick to these sentences..**
-L306: egg granulomas. **Reply: Amended in text, Tables, and legends to Figures and Tables with heartfelt thanks.**
-Fig 3: The illustrations are completely inadequate to corroborate the statement in L272. **Reply: The number and diameter of the egg granulomas distinctly differ in every section examined as shown in the representative Figure 3.**

-L319: correction of the position of the labeling. **Reply: Done with thanks. Figure 4 and Figure 5 too**
-L358 Il-17 ) or } and Protein or proteins. **Reply: Amended with thanks.**
-Fig.6: Nothing is seen on the pictures. **Reply: This histochemistry test was performed 4x times in view of obtaining clearer pictures and the figure now moved to Supplementary materials (S3 Fig).**

-L435: Who recommended this? **Reply: That was the conclusion of our previous paper with adjuvant-free MAP immunization [11].**
-L457: Increase. **Reply: Done with thanks, and change red marked.**
-L457: Please explain FhCL1. **Reply: (*Fasciola hepatica* cathepsin L1 as shown at the beginning of the Introduction.**

Reviewer #3: (No Response)

<b>Summary and General Comments</b></br></br>
Use  this section to provide overall comments, discuss strengths/weaknesses of the study, novelty, significance, general execution and scholarship. You may also include additional comments for the author, including concerns about dual publication, research ethics, or publication ethics. If requesting major revision, please articulate the new experiments that are needed.

**Reviewer #1:** This manuscript presents an investigation into the protective potential of a vaccine comprised of multiple antigen peptide constructs designed based on the sequences of parasite cathepsins and delivered with and without alum as an adjuvant. The results presented suggest these vaccine candidate resulted in a significant reduction in worm burden following a challenge infection with S. mansoni in outbred mice. The combination of MAP-1, MAP-2 and alum led to the most significant reduction in worm burden and the smallest size of intestinal granuloma. Some measures of immune response (cytokines, ROS) were measured in both liver and intestine of all animals.
The protection achieved in the vaccinated animals is remarkable and builds on the previous reports from this group regarding the efficacy of cysteine peptidases in anti-helminth vaccines.

I would suggest that the data could be presented differently. I am not sure that Fig 1 is necessary as this data would be better placed within Table 1. Similarly, the data presented in Fig 2 should be incorporated into Table 2.

**Reply: The Eminent Reviewer concern is correct: the upper panel of Figure 1 is redundant and was removed. The lower panel was kept as it shows data, which are not shown in Table 1, regarding worm fecundity.**

**Data in Figure 2 may certainly be added to Table 2, but are kept to illustrate the core of the problem and the article: that is the striking contrast between the cysteine peptidases-based vaccine highly significant, reproducible and commendable reduction in challenge worm burden associated with persistent lack of reduction, if not increase, of liver and small intestine worm burden. This is a serious vaccine flaw, and the reason was not intuitively clear. The liver cytokine findings implicating the IL-1 dual roles clarified the enigma and led to the conclusion: a vaccine composed uniquely of gut cysteine peptidases is not viable.**

The authors then performed a number of immunological assays to correlate with the protective effect of the vaccines. This section of the manuscript was very difficult to read as the data is presented in a variety of different formats.
•       Figure 4E – I would suggest that the treatment names are added to the lower axis of the graph rather than as a separate line of text – it is difficult to read as it is currently presented.

**Reply: Done as requested with thanks.**

•       Fig 5 – each of these graphs should be presented as a histogram with the treatment groups added to the lower axis – similar to the current presentation of Fig 4A.

**Reply: Thanks for this excellent advice. The data of Figure 5 are now perfectly clear and easy to follow.**
•       How many mice are presented in Fig 5? This information needs to be added to the legend.

**Reply: Five to eight and this information is now added to the Figure Legend.**
•       Although the statistical significance of data is mentioned in the text for Figure 5, there is no addition of significance markers to the graphs – can these please be added.

**Reply: All data were statistically analyzed in comparison to naïve, infected and alum-infected mice, and most differences were very clear, and mentioned in the text. Therefore, asterisks indicating statistical differences were added to the Figure only when necessary in order to avoid cramming a Figure of 10 panels with six columns each.**

In the descriptive text for the cytokine analysis there is multiple mentions of correlation between worm burden and cytokines – however, I would argue that these statements do not hold as if this was indeed the case there should be true correlation across all the treatment groups.  For example there is a claim that the increase in liver IL-1 correlated with the reduced worm burden – but if this was the case, then the MAP-1+ MAP-2 +Alum, which had the lowest worm burden, should have the highest IL-1, but the levels in this group look very similar to the infected cohort.
•       There are also a number of mechanistic conclusions drawn in the text (for example: led to modification of the host innate and acquired immune responses) without appropriate analysis being performed of relevant data presented.
•        I would suggest that the authors significantly temper the language and conclusions they are drawing from this data.

**Reply: The language and conclusions were tempered in the Results, Discussion and Conclusions, and changes red marked.**

Figure 6A – there is no need for both a table and a graph here – one or other will suffice. The graph would be a preferred option, although the axis labels needs to include the treatment groups and the associated statistical significance markings need to be added to the graph

**Reply: Figure 6A (now Fig 6) issues are amended as recommended: The Table removed, the significance values retained in the Legend to the Figure, and treatment groups added to the Figure.**

Figure 6B is quite unclear and perhaps redundant given the quantified assay presented in Figure 6A?

**Reply: Figure 6B is now moved to Supplementary Materials (S3 Fig) because Data Not Shown is no more allowed**.

The discovery that the vaccinated animals had significantly increased Arachadonic acid compared to the infected controls is interesting; however these animals also had significantly increased levels of ROS in their livers which appears slightly contradictory – although this is partly addressed in the discussion of the paper.

However, the discussion is a little hypothetical and a large portion of the theories presented seem to depend on the levels of IL-1 found within the liver – which as I mentioned previously seems to be variable affected by the vaccine preparations and is not a clear correlate with protection, or with the increased levels of ARA and ROS

The authors claim to have uncovered the immune mechanisms that mediate the protective effect (i.e IL-1, ROS and ARA) – however to support these claims some more direct mechanistic analysis would be required – again, I would suggest the authors apply a more measured approach to their discussion and conclusions.

The writing needs to be edited as there are a number of issues with the English, and sentence structure, throughout making parts of the text difficult to understand.

**Reply: The language and conclusions were tempered in the Results, Discussion and Conclusions, and changes red marked. The English language and style were thoroughly revised.**

**Reviewer #2:** Given the high relevance of the study, vaccines are urgently needed, and given the expert status of the authors, this study disappoints because of the poor quality of expression, language, and presentation. It appears that a prefinal version was submitted.

**Reply: Thanks are due to the Eminent Reviewers we hope will be satisfied with the revised version.**

Reviewer #3: The paper represents one in a long series of similar reports by this team pursuing cysteine proteases as vaccines against schistosomes, all are difficult to follow. While I agreed with the studies and outcomes these papers have seemed very similar and no taking big enough steps to warrant publication as a major paper. The authors should keep a focus and report only the most important data instead of rambling on about mechanisms of protection that are not fully elucidated here

**Reply: The language and conclusions were tempered in the Results, Discussion and Conclusions, and changes red marked. The English language and style were thoroughly revised.**

- the perfect cytokine balance is not known

**Reply: YES, and perfect balance is now omitted..**

The figures are poorly prepared and presented - are they all needed? Could they just not be added to supplementary section or simple referred to data not shown.

**Reply: Text and Figures were thoroughly revised and amended. Unneeded figures were removed (e.g. upper panel of Fig. 1) or added to supplementary section (Fig 6B now S3).**

The paper isn't making a step-change

Reply: **The article answers a problem long associated with the cysteine peptidase-based schistosomiasis vaccine: that is the striking contrast between the highly significant, reproducible and commendable reduction in challenge worm burden associated with persistent lack of reduction, if not increase, of liver and small intestine worm burden. This is a serious vaccine flaw, and the reason was not intuitively clear. The liver cytokine findings implicating the IL-1 dual roles clarified the enigma and led to the conclusion: a vaccine composed uniquely of gut cysteine peptidases is not viable. The article furthermore provided the solution involving incorporating a peptide derived from a lung-stage larvae ESP to target both the lungs and liver. As a matter of fact we run for a preliminary experiment mixing MAP-1 with thioredoxine peroxidase-derived MAP, and were blessed to tears with significant (*P* = 0.0079) egg burden reduction of 65.4% in liver and 75.9% in small intestine, for the first time after so many trials and experiments and publications. More experiments are set to confirm the findings and evaluate the underlying immune responses.**

and in my opinion is best suited to a vaccine-related journal.

**Reply: PNTD is hailed for supporting the different scientific aspects of NTD, on top of which are certainly the importance and problems of vaccine development.**

Some questions –

1. Why exactly were these short peptide chosen.

**Reply: As stated in the Introduction "peptide sequences shared by these cysteine peptidases may substitute for the whole molecule and form the basis of a safe, cost-effective, chimeric protein vaccine, easy to manufacture and deliver in countries with limited resources". The peptide sequences were selected based on extensive sharing with schistosome gut cysteine peptidases and papain as detailed in reference [11] and not repeated for fear of self-plagiarism.**

Do they also match with host cysteine protease and have the potential to induce auto-immune responses?

**Reply: Matching with host cysteine proteases leads to limited immune, especially antibody, responses to the schistosome gut cysteine peptidases immunogens as observed in this and previous articles, and precludes autoimmune responses, as added now in the Introduction, lines 68-70, and red marked.**

2. Why was MAP chosen rather than linking the peptides to another carrier? Would they work with another carrier?

**Reply: MAP constructs were chosen because of their ability to stimulate higher immune responses than linear peptides, and to avoid involving any other molecule.**

3. Wasn't sure of the meaning in 74 - 76 sentence - are they authors saying that the antibodies to peptides do not block the enzymes activity. If not how do they block the enzyme function.

**Reply: This sentence was deleted**

4. line 110 - 39 rather than 40?

**Reply: Amended with thanks. We usually use and extra mouse in the control group and that issue now amended and red marked.**

5. Line 125 - not sure why egg response would interfere with peptide cytokine responses.  This statement is not clear.

**Reply: The egg antigens are numerous and strongly immunogenic. They induce immune responses able to confound reactivity to every immunogen, especially short peptides.**

--------------------
