# Supplementary material for: Increased hepatic interleukin-1, arachidonic acid, and reactive oxygen species mediate the protective potential of peptides shared by gut cysteine peptidases against Schistosoma mansoni infection in mice
Source: PLoS Negl Trop Dis. 2023 Mar 15;17(3):e0011164. doi: 10.1371/journal.pntd.0011164 (PMC10042345; doi:10.1371/journal.pntd.0011164)
Supplement: Attachment — Submitted filename: Response to Reviewer 1.docx [file pntd.0011164.s005.docx]

Response to Reviewer #1

However, in the results section describing the measure of cytokines in tissue (around Line 305) a number of statements which claim correlations between specific cytokines and a protective effect remain. These are hypothetical and as such belong in the discussion (where this is addressed) and should only be in the results section of actual statistical correlations have been performed. This section of next needs to be edited accordingly such that only the actual results are described here and not a proposal as to the function/outcome of these

**Reply: Agreed with thanks and as required, four sentences in the Results section were deleted and changes marked in red.**

There remains some minor issues with the English in parts (although improved greatly since the original submission) - I would suggest a native english speaker performs an edit/read **through prior to resubmission.**

**Reply: The English style and grammar were again thoroughly revised by a native English Speaker**.

With sincere thanks and kind regards

Hatem Tallima and Rashika El Ridi
